# Psychological distress following a motor vehicle crash: preliminary results of a randomised controlled trial investigating brief psychological interventions

**DOI:** 10.1186/s13063-018-2716-2

**Published:** 2018-06-27

**Authors:** Rebecca Guest, Yvonne Tran, Bamini Gopinath, Ian D. Cameron, Ashley Craig

**Affiliations:** John Walsh Centre for Rehabilitation Research, Kolling Institute of Medical Research, Sydney Medical School-Northern, The University of Sydney, Corner Reserve Road and First Avenue, Royal North Shore Hospital, St Leonards, NSW 2065 Australia

**Keywords:** CBT/cognitive behaviour therapy, Depression, Anxiety, PTSD/post-traumatic stress disorder, MVC/motor vehicle crash, Injury

## Abstract

**Background:**

The preliminary results of a randomised controlled trial are presented. The aim of the trial is to determine the efficacy, feasibility and acceptability of email-delivered psychological interventions with telephone support, for adults injured in a motor vehicle crash engaged in seeking compensation. The primary intention for this preliminary analysis was to investigate those who were psychologically distressed and to stop the trial midway to evaluate whether the safety endpoints were necessary.

**Methods:**

The analysis included 90 adult participants randomised to one of three groups, who were assessed at baseline and post-intervention at 3 months. Cognitive behaviour therapy (CBT) or healthy lifestyle interventions were delivered over 10 weeks, involving fortnightly emailed modules plus clinically focussed telephone support, with the aim of reducing psychological distress. An active waiting list of control subjects received non-clinically focussed telephone contact over the same period along with claim-related reading material. Depression Anxiety Stress Scales (DASS) and Impact of Events Scale (Revised) (IES-R) were used to assess psychological distress. Psychiatric interviews were used to diagnose major depressive disorder and post-traumatic stress disorder. Aspects of acceptability and feasibility were also assessed.

**Results:**

For those diagnosed with depression at baseline in the CBT group, psychological distress reduced by around 16%. For those with depression in the healthy lifestyle group, distress increased marginally. For those in the control group with depression, distress also decreased (by 18% according to DASS-21 and 1.2% according to IES-R). For those without depression, significant reductions in distress occurred, regardless of group (*P* < .05). The results suggest that for those with depression, a healthy lifestyle intervention is contraindicated, necessitating the cessation of recruitment to this intervention. The interventions were reported as acceptable by the majority and the data indicated that the study is feasible.

**Conclusions:**

CBT with telephone support reduced psychological distress in physically injured people with depression who are engaged in seeking compensation. However, time plus fortnightly telephone contact with claim-related reading material may be sufficient to reduce distress in those who are depressed. For those who were not depressed, time plus telephone support is most likely sufficient enough to assist them to recover. The trial will continue with further recruitment to only the CBT and control groups, over longer follow-up periods.

**Trial registration:**

Australian and New Zealand Clinical Trials Registry: Preventing psychological distress following a motor vehicle accident; ACTRN12615000326594. Registered on 9 April 2015.

**Electronic supplementary material:**

The online version of this article (10.1186/s13063-018-2716-2) contains supplementary material, which is available to authorized users.

## Background

Psychological distress is elevated in those who have experienced a motor vehicle crash (MVC) [1–5]. The psychosocial impacts of non-catastrophic injuries sustained in MVCs can be debilitating and include chronic pain, disability, loss of income, trauma and stress placed on relationships [5, 6]. The risk of a psychological disorder, such as major depressive disorder (MDD) and post-traumatic stress disorder (PTSD), is also high [1, 7]. Furthermore, involvement in seeking MVC-related compensation is associated with increased levels of psychological distress compared to those not claiming [8]. Research has shown that psychological distress and rates of MDD and PTSD remain elevated up to at least 12 months post-MVC [5, 9].

Given the above, a strategy for reducing the risk of psychological distress would involve providing brief interventions to MVC survivors engaged in seeking compensation as soon after the MVC as practicable [10–12]. Potential interventions include cognitive behaviour therapy (CBT) involving, for example, psychoeducation about the dynamics of distress and injury, stress reduction and helpful thinking techniques, the goal being to enhance adaptive psychological, social and behavioural skills. Another strategy involves healthy lifestyle (HL) interventions focussing on sleep, diet and exercise [13–15].

CBT is an efficacious treatment for disorders such as MDD and PTSD [11, 15–19]. Likewise, HL interventions have been shown to increase well-being [14, 15, 20], and there is growing support for improvements in mental health associated with increased regular physical activity and improved sleep [13, 21–25].

The internet provides a novel platform for the delivery of psychological interventions, by way of an online biblio-therapy with therapist contact [18, 26]. Evidence exists for the efficacy of this style of delivery for lifestyle and psychological disorders [14, 27]. Further, a systematic review identified that an online-delivered CBT program was as effective as face-to-face CBT in reducing depressive symptoms [18, 28, 29]. Email delivery has the advantage of no web-based costs, and arguably results in personable delivery of CBT, especially if clinically focussed telephone support is provided. If the trial shows the benefit of such brief interventions, internet-based modes of delivery could be explored.

Given the very limited research into the management of psychological distress in MVC compensation claimants [11], there is a need for controlled research into the efficacy of strategies designed to lower risk of distress and disorder in this area. Therefore, adults who experienced a non-catastrophic injury in an MVC and were engaged in seeking compensation were invited to participate this study. The goal was to determine the efficacy of CBT and HL interventions, integrated with clinically focussed telephone support, to reduce psychological distress compared to a control group of people with an MVC injury engaged in seeking compensation. Whilst it is expected that the majority of people will recover regardless of the intervention [5], we believe that those who have elevated psychological distress at baseline will be more complex to treat and require careful monitoring. Given the vulnerability of those with elevated psychological distress, the primary intention of the preliminary analysis was to compare those with a baseline diagnosis of MDD versus those without a diagnosis of MDD. A further primary intention was to determine the necessity of implementing the safety endpoints for either of the interventions, should the results show any degree of harm. It was hypothesised that those without MDD would show improvement at the 3-month assessment, with the CBT and HL groups showing superior improvement. It was also hypothesised that those with MDD in the CBT and HL groups would show significantly reduced distress compared to the control group.

## Methods

### Participants

**Fig. 1:**
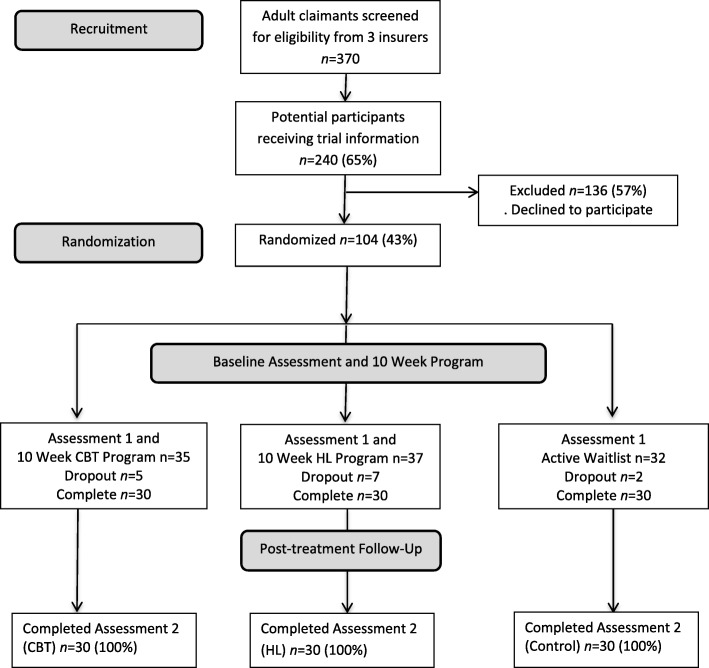
Consort flow diagram. CBT cognitive behaviour therapy, HL healthy lifestyle

The details of the protocol, such as recruitment, design, setting and aims for this randomised controlled trial (RCT), have been previously reported in the study’s published protocol paper [30]. A CONSORT checklist file shows how the recommendations for a clinical trial have been addressed (Additional file 1). Participants included MVC survivors who lodged a compensation claim in New South Wales (NSW) or Victoria, Australia, between July 2015 and May 2017. Subsequent to publication of the protocol paper [30], a third recruitment site was secured in NSW. Recruitment sites included: (1) Suncorp and (2) the National Roads and Motorists’ Association in NSW and (3) the Transport Accident Commission in Victoria. Figure 1 shows the flow for the study’s recruitment, intervention and assessment processes. The NSW compulsory third-party insurance system is fault based; therefore, none of the NSW people in the study were at fault in their crash, whereas Victoria participants could be either at fault or not at fault. The inclusion criteria were (1) adult (age > 18 years) survivor of an MVC who lodged a claim within 4 months of their MVC and (2) English speaking. Exclusion criteria included the presence of severe injuries, such as spinal cord injury, amputation, blindness, severe traumatic brain injury and other injuries requiring extended hospitalisation. Table 1 shows participant characteristics including socio-demographic, injury and intervention-related characteristics by group.

**Table 1:** Participant characteristics by group including demographic, injury and intervention-related characteristics

Characteristics	Control	HL	CBT	Total
Age: mean years (SD)	47.67(14.8)	47.83(13.2)	42.70(16.0)	46.07(14.7)
Female *n* (%)	21(70.0)	19(63.3)	21(70.0)	61(67.8)
BMI *M* (SD)^a^	26.31(5.5)	29.74(6.0)	28.90(9.1)	28.27(7.2)
Marital status *n* (%)				
Single	5(16.7)	4(13.3)	8(26.7)	17(18.9)
Widowed	0(0.0)	1(3.3)	2(6.7)	3(3.3)
Divorced/separated	7(23.3)	3(10.0)	9(30.0)	19(21.1)
Married/de facto	18(60.0)	22(73.4)	11(36.6)	51(56.7)
Education *n* (%)				
Up to year 10	8(26.7)	5(16.7)	5(16.7)	18(20.0)
Year 12 or equivalent	3(10.0)	4(13.3)	3(10.0)	10(11.1)
Technical or further education	9(30.0)	3(10.0)	7(23.3)	19(21.1)
University	10(33.3)	18(60.0)	15(50.0)	43(47.8)
Work *n* (%)				
Full time	16(53.3)	14(46.7)	17(56.7)	47(52.2)
Part time	8(26.8)	8(26.7)	7(23.3)	23(25.6)
Pensioner	4(13.3)	7(23.3)	4(13.3)	15(16.7)
Unemployed	1(3.3)	1(3.3)	2(6.7)	4(4.4)
Student	1(3.3)	0(0.0)	0(0.0)	1(1.1)
Role in MVC				
Driver	13(43.3)	21(70.0)	18(60.00)	52(57.8)
Passenger	5(16.7)	6(20.0)	2(6.66)	13(14.4)
Motorbike rider	5(16.7)	0(0.0)	8(26.66)	13(14.4)
Bicyclist	3(10.0)	3(10.0)	1(3.33)	7(7.8)
Pedestrian	4(13.3)	0(0.0)	1(3.33)	5(5.6)
Days since MVC: *M* (SD)	96.77(71.8)	81.13(52.3)	68.37(41.2)	82.09(57.1)
Days in hospital: *M* (SD)	0.80(1.7)	1.37(4.2)	0.93(1.8)	1.03(2.8)
Injury type/location				
Neck *n* (%)	5(17.2)	11(36.8)	8(28.6)	24(27.6)
Shoulder *n* (%)	4(13.8)	1(3.3)	2(7.1)	7(8.1)
Arm *n* (%)	2(6.9)	1(3.3)	2(7.1)	5(5.7)
Upper back *n* (%)	3(10.3)	4(13.3)	4(14.3)	11(12.6)
Lower back *n* (%)	7(24.1)	3(10.0)	4(14.3)	14(16.1)
Leg *n* (%)	6(20.8)	6(20.0)	4(14.3)	16(18.4)
Head *n* (%)	0(0.0)	3(10.0)	1(3.6)	4(4.6)
Chest/abdomen *n* (%)	2(6.9)	1(3.3)	3(10.7)	6(6.9)
Pain intensity *M* (SD)^b^	7.10(1.9)	6.73(2.8)	6.30(2.5)	6.71(2.4)
Perceived danger in MVC *M* (SD)	2.87(1.3)	3.30(1.5)*	2.37(1.1)	2.84(1.4)
None or small: *n* (%)	12(40.0)	11(36.7)	16(53.3)	39(43.3)
Moderate, great, overwhelming: *n* (%)	18(60.0)	19(63.3)	14(46.7)	51(56.7)
Physical Health Composite Index: *M*(SD)	33.77(7.3)	34.36(10.1)	36.54(9.3)	34.89(9.0)
Mental Health Composite Index: *M*(SD)	42.09(14.0)	41.74(15.4)	44.79(14.3)	42.88(14.5)
Treated by psychologist/psychiatrist pre-MVC				
*n* (yes)	6(20.00)	7(23.3)	15(50.0)	28(31.1)
*n* (no)	24(80.00)	23(76.7)	15(50.0)	62(68.9)
Psychiatric medications pre-MVC^c^				
*n* (yes)	8(26.7)	6(20.0)	8(26.7)	22(24.4)
*n* (no)	22(73.3)	24(80.0)	22(73.3)	68(75.6)
Intervention-related characteristics				
Telephone calls *M* (SD)^d^	2.47(1.6)	3.27(2.8)	3.23(2.6)	2.99(3.3)
Quality of rapport *n* (%)^e^				
Poor	5(16.7)	7(23.33)	2(6.7)	14(15.6)
Somewhat	11(36.7)	10(33.3)	10(33.3)	31(34.4)
Excellent	14(46.7)	13(43.3)	18(60.0)	45(50.0)
Email versus postal delivery *n* (%)				
Email	28(93.3)	27(90.0)	29(96.7)	84(93.3)
Postal	2(6.7)	3(10.0)	1(3.3)	6(6.7)

*BMI* body mass index, *CBT* cognitive behaviour therapy, *HL* healthy lifestyle, *M* mean, *MVC* motor vehicle crash, *SD* standard deviation

*HL group significantly different to CBT (*P* < .05)

^a^Data missing for three people for HL; total *n* = 87

^b^Pain intensity score from 1 to 10

^c^Medication information was obtained with the question ‘Have you ever been prescribed medication for anxiety or depression?’

^d^Telephone calls relates to the number of calls required by the researcher to achieve the participants’ second assessment

^e^Quality of rapport between researcher and participant is a subjective rating by the researcher who made the telephone calls

### Design

This RCT is ongoing, with participants being assessed across four time points: baseline assessment upon entry into the RCT (mean 12 weeks post-MVC), and 3, 6, and 12 months post-intervention. For this preliminary investigation, the trial was stopped at the midway point when 30 participants in each group had completed the baseline, the intervention and the 3-month post-intervention assessment.

### Sample size

Based on prior results for the CBT and HL interventions [7, 14, 15], the two interventions were assumed to have at least a small to moderate mean effect size of 0.25 (Cohen’s *d*) compared to the control group. Assuming α = 0.05 and a power of 80% for a three-group comparison analysis with four measures over time, a sample of 135 participants needed to be recruited to detect true differences [30]. It was, therefore, proposed to recruit at least 180 participants to accommodate loss to follow-up of approximately 25% based on similar research [31].

### Procedure

The preliminary analysis shows a total of 240 individuals received information about the trial, of whom 104 were randomised into one of three groups: CBT (*n* = 37), HL (*n* = 35) or active waiting list (control, *n* = 32). We included 104 participants in this analysis to ensure attrition did not impact on the 30/30/30 preliminary analysis. The CBT and HL groups received fortnightly modules in Microsoft PowerPoint presentation format with homework worksheets, self-monitoring templates and instructions for using CBT or HL skills. CBT and HL participants also received clinically focussed telephone support on alternate weeks to encourage and remind them to practise the skills and to read the module material. The control group received compensation-related material every second week and a phone call on the alternate week. The reading material for the control group was restricted to publicly accessible claim information, and telephone calls confirmed this information was received. Table 2 shows the content of each module for each intervention group. Preliminary findings for the first 90 participants who completed the baseline assessment, interventions and 3-month follow-up are reported here and Fig. 1 shows the flow of these participants from eligibility through to this preliminary analysis.

**Table 2:** Contents of each of the five modules for CBT, lifestyle and waiting list control groups

	CBT	Lifestyle	Control
1	Overview	Overview	Reading; A guide for people injured in an MVC
2	Mood and slow breathing skills, self-monitoring	Goal-setting and life-pacing	Reading: Overview of the claims process
3	The art of distraction, applying distraction	Self-monitoring and changing unhelpful behaviours	Reading: Obligations of the insurer
4	Stress and helpful thinking, evidenced-based thinking	Sleep hygiene and sleep self-monitoring	Reading: Obligations of the claimant
5	Problem-solving and conclusions, self-mastery, changeable vs. unchangeable problems	Improving well-being through diet and physical exercise, social participation, daily activity schedule	Reading: Finalising a claim

*CBT* cognitive behaviour therapy, *MVC* motor vehicle crash

### Measures

A selection of the measures employed in this study have been analysed for this preliminary investigation. These include socio-demographics, MVC and injury characteristics, pre-injury mental health, self-reported body mass index (BMI), pain intensity and aspects of acceptability and feasibility. Psychometric measures included the Depression, Anxiety and Stress Scale (DASS-21) and the Impact of Events Scale (Revised) (IES-R). Structured interviews based on criteria from the 5th Edition of the Diagnostic and Statistical Manual of Mental Disorders (DSM-5) were used to diagnose MDD and PTSD.

DASS-21 is a 21-item self-report scale providing an assessment of the severity of psychological distress as a total score and in three domains: depressive mood, anxiety and stress [32, 33]. Participants completed 21 4-point Likert items (0–3) assessing self-reported distress over the past week. Higher scores indicate elevated distress. Total scores are calculated by summing all 21 items [33], and then, in accordance with the DASS-42, scores were multiplied by 2 [33]. DASS-21 has sound psychometric properties, including acceptable internal reliability and validity [32].

IES-R is a 22-item self-report measure of trauma-related distress [34], validated in people with traffic injuries [35]. Respondents indicate their degree of distress during the past 7 days related to their recent MVC. It is a 5-point scale ranging from 0 (not at all) to 4 (extremely) with subscales for avoidance (e.g. avoidance of feelings or situations), intrusion (e.g. distressing thoughts) and hyperarousal (e.g. irritability and hypervigilance). Domains are scored by determining the mean item score [34]. Higher scores indicate increased distress.

#### DSM-5

MDD was diagnosed by clinician interview if participants met relevant DSM-5 criteria by answering the relevant questions on their online assessments. All participants had experienced trauma in their recent MVC, satisfying the first requirement for a PTSD diagnosis [36]. They also needed to report at least one of the essential PTSD symptoms described by DSM-5.

#### Short Form 12

The Short Form 12 (SF-12) includes 12 items from the SF-36 [37] self-report health survey. It produces the physical component summary and the mental component summary scores. Scoring of items is identical to the SF-36 and higher scores mean a higher quality of life.

#### Acceptability and attrition

The acceptability of the interventions was measured post-treatment for the CBT and HL groups using four questions: 0

1. Overall, how satisfied were you with the program?
2. How satisfied were you with the modules and module summaries?
3. Would you feel confident in recommending this treatment to a friend?
4. Was it worth your time doing the program?

Participants responded to the first two questions using a 5-point Likert scale, with choices ranging from ‘very satisfied’ to ‘very dissatisfied’, while the second two questions used a ‘yes’ or ‘no’ response. These questions have been used successfully in prior research to examine the acceptability of internet-delivered CBT [38, 39]. Attrition relates to the total number of participants who dropped out prior to the second measure.

#### Feasibility

Feasibility was determined by analysing (1) the percentage of people offered the interventions versus those who declined, (2) the time from consent to commencement in the study, (3) the percentage attrition from randomisation through to post-treatment assessment and (4) the ease at which email delivery is available to participants by comparing the number of email-delivered versus post-delivered programs.

### Data analysis

Participants were given an identification number to ensure anonymity, and an internet link to complete their assessments through Survey Gizmo, a professional online secure survey software tool. All groups completed identical assessments at baseline and post-intervention (approximately 3 months post-injury). The data analyst had no contact with participants and was blind to randomisation. The intervention program coordinator, who also provided clinically focussed telephone support, was blind to all assessment data.

The purpose of this preliminary analysis was to analyse participants diagnosed with MDD at baseline and determine whether cessation of recruitment to either intervention group was required. As part of this analysis, preliminary efficacy trends were investigated to provide some indication of acceptability and feasibility. Statistical significance at this point in the RCT analysis was not the major objective, nevertheless limited statistical analyses were performed using SPSS version 22 [40]. While it is understood these analyses were preliminary and underpowered, we believe it is important to examine efficacy trends. The analysis included descriptive statistics for the primary outcome measures (Table 3). General linear repeated measures MANCOVA was used to determine differences over time and between groups for the total scores from DASS-21 and IES-R. The total scores were used as primary outcomes as they provide maximum variance. As described in the original protocol [30], it was decided to adjust for variables believed to influence primary outcomes (e.g. the baseline value of a primary outcome) and those pre-specified in the protocol [41]. Therefore, as specified, physical health (the physical component summary of the baseline SF-12) was adjusted for in the DASS-21 and IES-R analyses. Further, baseline DASS-21 depressive mood was adjusted for in the DASS-21 total score analyses and IES-R intrusion was adjusted for in the IES-R total score analysis. These two baseline measures were chosen because they are related to the primary outcome and their inclusion as covariates that were adjusted for baseline severity improved the efficiency of the analyses. Baseline differences between groups were regarded as randomisation anomalies [42].

**Table 3:** Results for pre- and post-intervention DASS-21 and IES-R by group

	Control	HL	CBT
	Mean (SD) 95% CI	Mean (SD) 95% CI	Mean (SD) 95% CI
DASS-21 baseline			
Depression	13.0(13.5) 7.9–18.0	15.9(13.5) 10.8–20.9	10.8(11.3) 6.6–15.0
Anxiety	11.6(12.6) 6.9–16.3	14.0(13.0) 9.1–18.9	9.7(10.4) 5.8–13.6
Stress	17.5(14.4) 12.2–22.9	18.9(13.6) 13.9–24.0	14.2(12.0) 9.7–18.7
Total	42.1(39.0) 27.6–56.7	48.8(37.7) 34.7–62.9	34.7(30.3) 23.3–46.0
DASS-21 3 months			
Depression	9.7(11.6) 5.3–14.0	15.8(14.1) 10.5–21.1	9.0(10.8) 5.0–13.0
Anxiety	8.7(9.8) 5.1–12.4	14.1(14.5) 8.7–19.5	7.5(8.5) 4.3–10.6
Stress	14.0(12.9) 9.2–18.8	16.7(13.2) 11.8–21.7	11.8(10.9) 7.7–15.9
Total	32.4(31.9) 20.5–44.3	46.7(39.9) 31.8–61.6	34.7(30.3) 23.3–46.0
IES-R baseline			
Intrusion	14.8(8.9) 11.5–18.2	18.4(9.3) 15.0–21.9*	12.1(9.4) 8.6–15.6*
Avoidance	11.8(8.8) 8.5–15.1	15.4(9.1) 11.9–18.7	9.9(7.6) 7.1–12.8
Hyperarousal	11.3(6.6) 8.9–13.8	13.3(7.5) 10.5–16.1	9.2(6.8) 6.6–11.7
Total	37.9(22.9) 29.4–46.5	47.1(23.9) 38.1–56.0*	31.2(22.1) 23.0–39.5*
IES-R 3 months			
Intrusion	12.7(9.1) 9.3–16.1	16.0(10.8) 11.9–20.0*	9.8(7.5) 7.0–12.6*
Avoidance	10.7(8.4) 7.5–13.8	13.7(9.7) 10.0–17.3*	8.0(7.4) 5.3–10.8*
Hyperarousal	9.4(7.8) 6.4–12.3	11.9(8.1) 8.9–15.0	7.3(6.0) 5.3–9.5
Total	32.7(24.0) 23.8–41.7	41.6(27.5) 31.3–51.8*	25.1(19.4) 17.8–32.4*

These are unadjusted descriptive scores only

*CBT* cognitive behaviour therapy, *DASS-21* Depression, Anxiety and Stress Scale 21, *HL* healthy lifestyle, *IES-R* Impact of Events Scales (Revised), *SD* standard deviation

*P < .05

## Results

### Baseline group characteristics and MDD or PTSD diagnosis

Table 1 shows that between groups, the participants did not differ significantly by age, sex, BMI, marital status, education, employment, role in the MVC, days since the MVC, injury type, perceived pain intensity or previous mental health history. Table 3 shows descriptive unadjusted data for DASS-21 and IES-R measures over time. No significant differences were found between groups for baseline DASS-21 and domains. For IES-R, the HL group had significantly higher levels of intrusion and total scores compared to the CBT group (*P* < .05). Table 4 shows the frequencies of diagnosed MDD and PTSD by groups over time. There was no significant difference in baseline rates of MDD, or over time between groups (χ^2^_2_ = 1.4; *P* > .05). There was a significant difference in baseline rates of PTSD (χ^2^_2_ = 9.5; *P* < .01; HL had a higher rate of PTSD). No group differences in rates of PTSD occurred post-intervention.

**Table 4:** Baseline and 3-month frequencies of MDD and PTSD by group according to DSM criteria

	Control	HL	CBT	Total
	*n* (%)	*n* (%)	*n* (%)	*n* (%)
MDD at baseline	15 (50.00)	18 (60.00)	13 (43.33)	46 (51.11)
MDD at 3 months	14 (46.66)	18 (60.00)	14 (46.66)	46 (51.11)
PTSD at baseline	2 (6.66)	11 (36.66)	4 (13.33)	17 (18.88)
PTSD at 3 months	8 (26.66)	14 (46.66)	9 (30.00)	31 34.44)

*CBT* cognitive behaviour therapy, *DSM* Diagnostic and Statistical Manual of Mental Disorders, *HL* healthy lifestyle, *MDD* major depressive disorder, *PTSD* post-traumatic stress disorder

### DASS-21 and IES-R total score primary outcomes

**Fig. 2:**
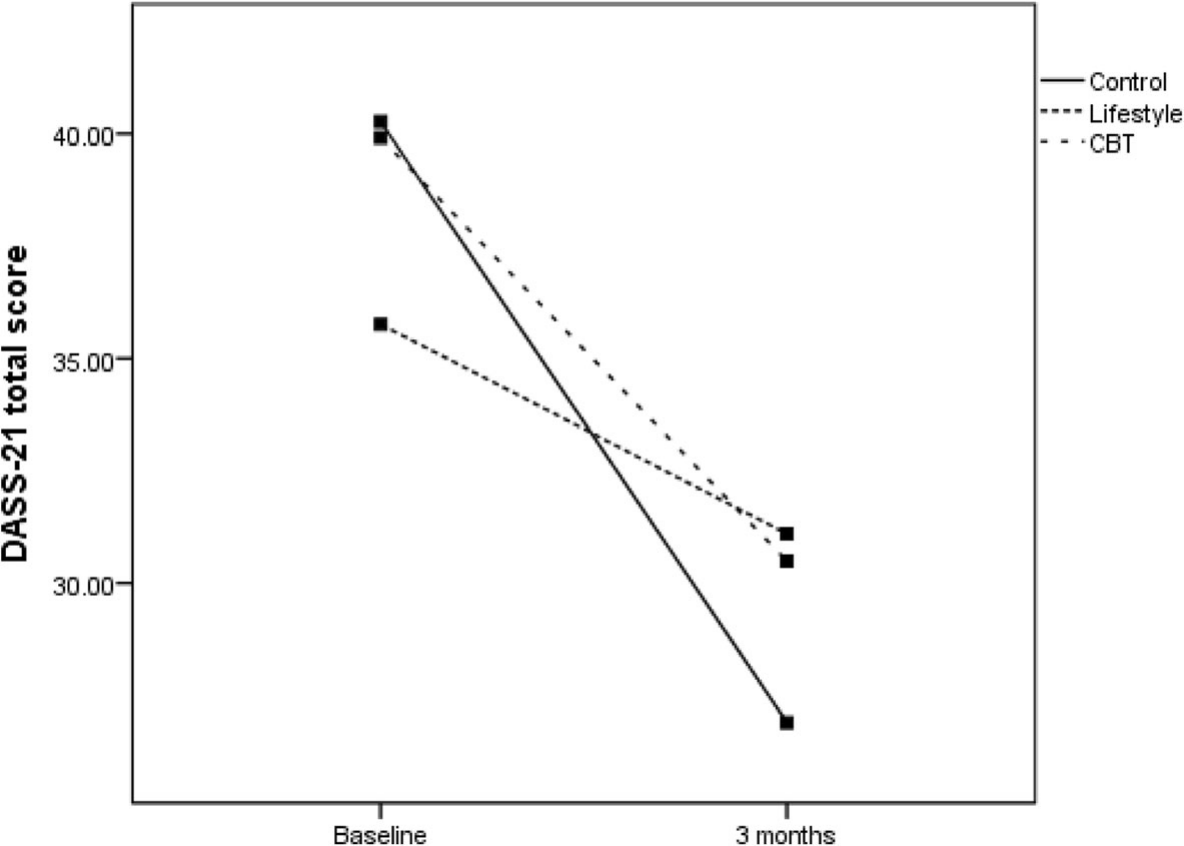
DASS total score for the three groups for those without a diagnosis of MDD. CBT cognitive behaviour therapy, DASS Depression, Anxiety and Stress Scale, MDD major depressive disorder

**Fig. 3:**
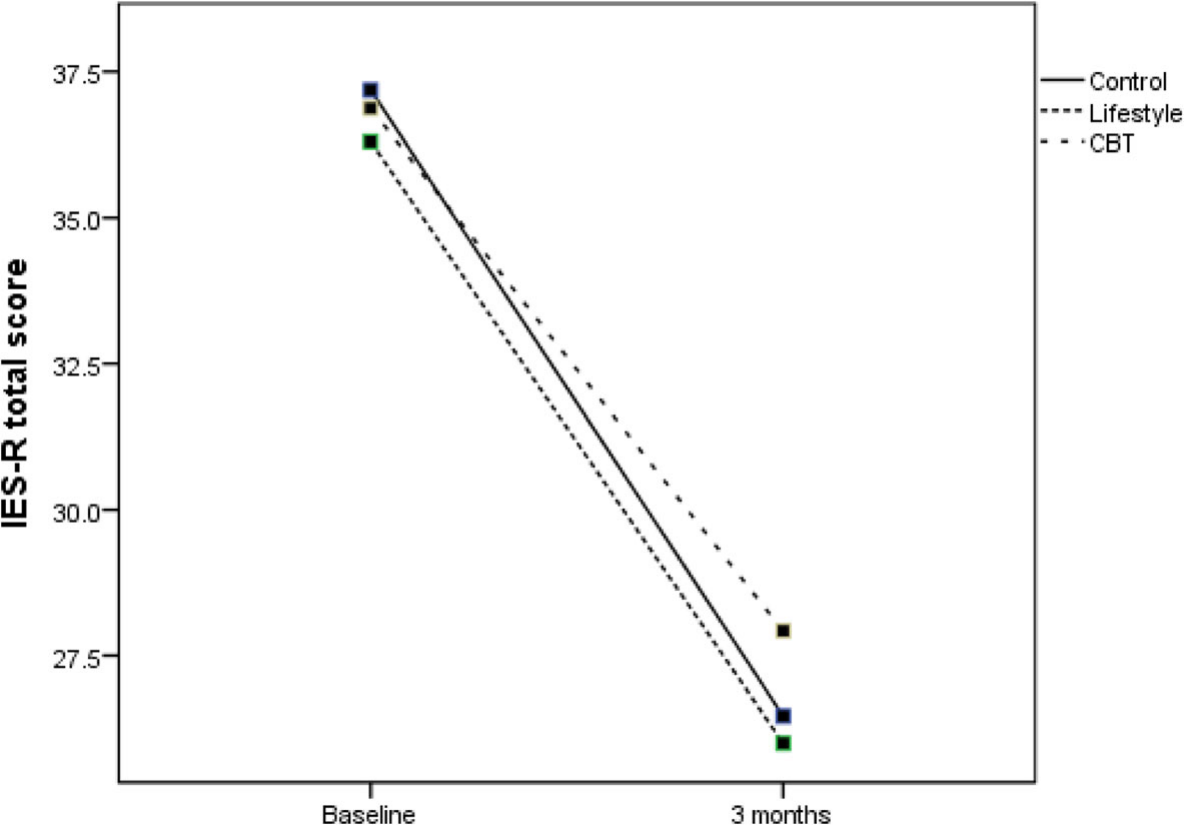
IES Total for the three groups for those without diagnosis of MDD. The analysis was adjusted for baseline SF12 physical health composite index and baseline IES Intrusion domain

**Fig. 4:**
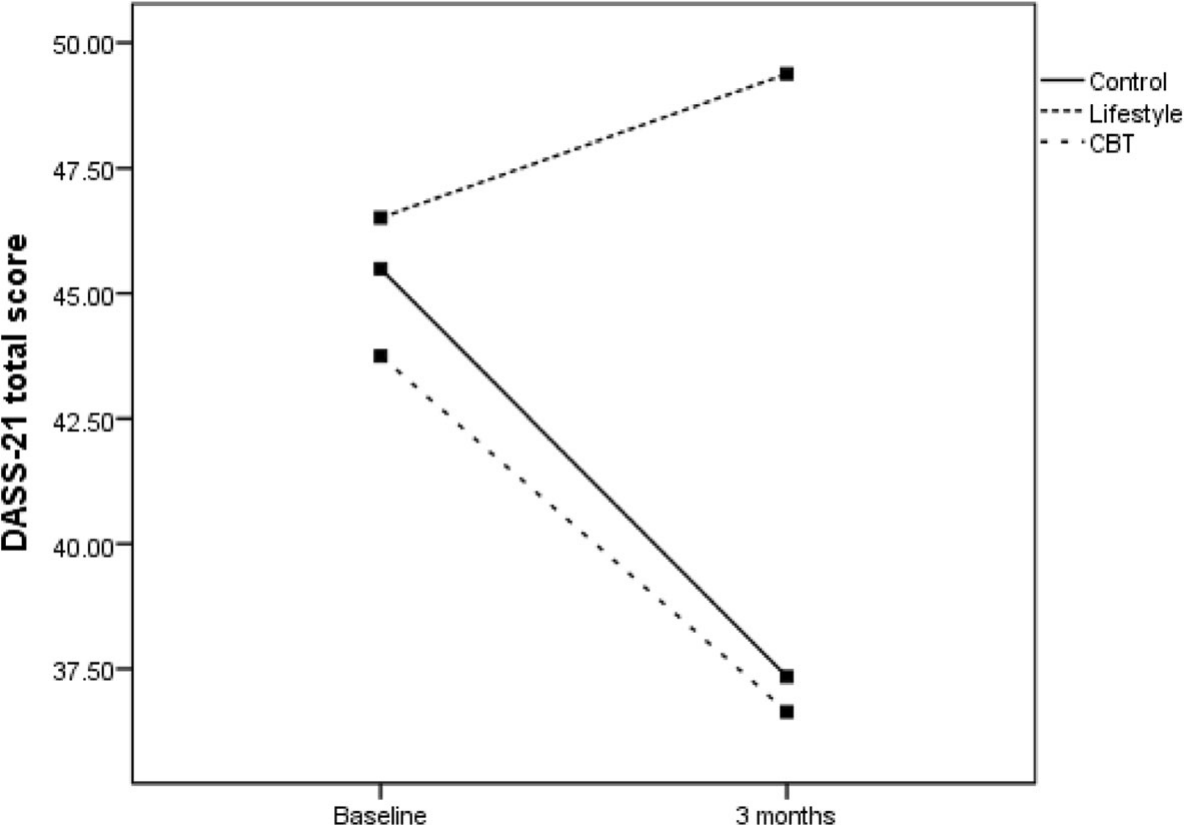
DASS total for three groups for those with diagnosis of MDD. The analysis was adjusted for baseline SF12 physical health composite index and baseline DASS depression domain

**Fig. 5:**
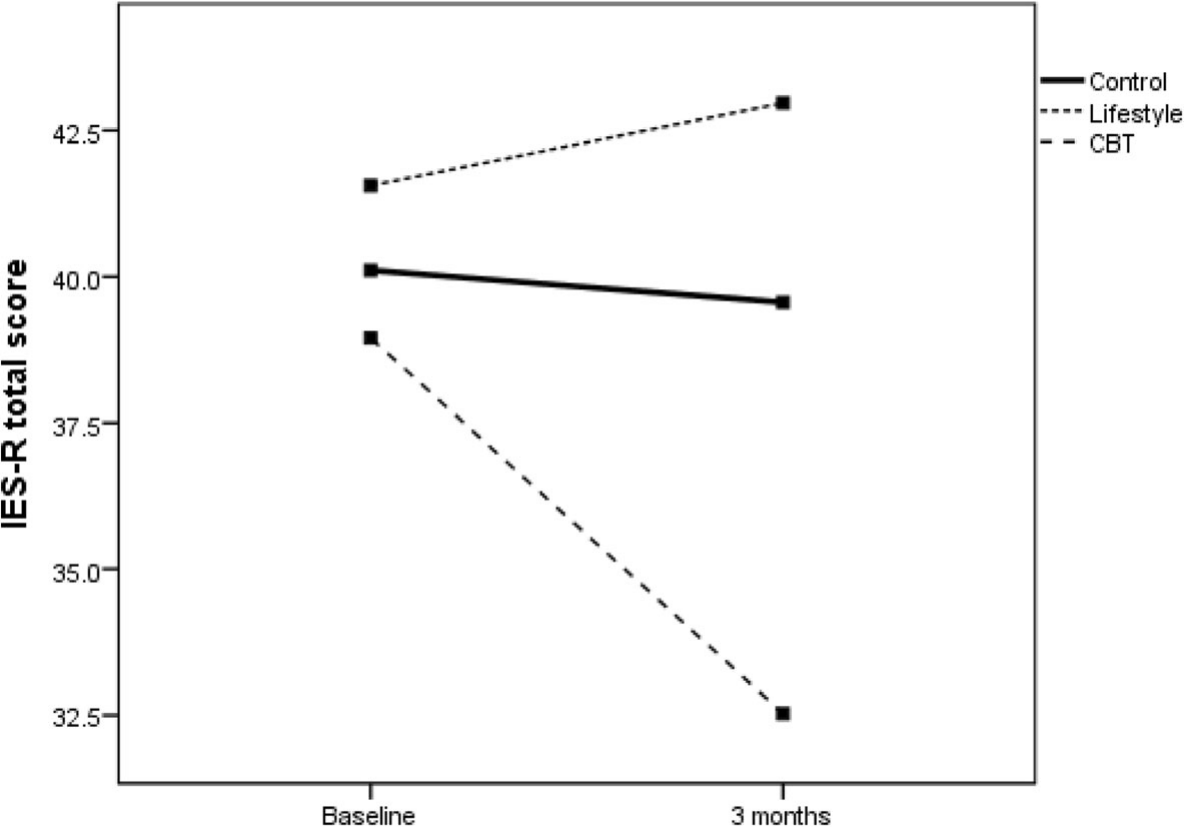
IES Total for the three groups for those with a diagnosis of MDD. The analysis was adjusted for baseline SF12 physical health composite index and baseline IES Intrusion domain

The primary sub-group analyses compared changes in the primary measures between groups and those meeting DSM-5 criteria for MDD (no separate sub-group PTSD analyses were conducted as all but one participant with PTSD met the criteria for MDD). For participants without MDD, Fig. 2 (DASS-21 total score) and Fig. 3 (IES-R total score) show significant reductions in distress over time for all groups as measured by DASS-21 (*F*(1,79) = 4.0; *P* < .05) and by IES-R (*F*(1,79) = 10.0; *P* < .01). For those diagnosed with MDD, Fig. 4 (DASS-21 total score) shows a non-significant interaction effect in which the CBT and control groups trend toward reduced distress over time compared to the HL group. For those with MDD, Fig. 5 (IES-R total score) shows a non-significant interaction effect in which the CBT group shows a trend toward reduced distress compared to the HL and control groups. Table 5 shows adjusted means for group by MDD status. Secondary analyses showed there was no main effect or interaction differences between total groups over time, though a significant reduction in distress occurred over time regardless of group for DASS-21 and IES-R (*P* < .01).

**Table 5:** Estimated marginal adjusted means for each group with and without MDD

	Control	HL	CBT
	Mean (SE) 95% CI	Mean (SE) 95% CI	Mean (SE) 95% CI
DASS-21			
Without MDD			
Baseline	40.27(3.3)33.7–46.8	35.76(3.8)28.2–43.3	39.92(3.1)33.8–46.0
3 months	26.90(7.1)12.7–41.1	31.10((8.2)14.8–47.4	30.40(6.6)17.4–43.6
With MDD			
Baseline	45.49(3.2)39.1–51.8	46.51(3.1)40.4–52.6)	43.75(3.3)37.3–50.2
3 months	37.34(6.9)23.7–51.0	49.38(6.6)36.3–52.5	36.65(7.0)22.7–50.7
IES-R			
Without MDD			
Baseline	37.18(2.2)32.8–41.6	36.31(2.5)31.3–41.4	36.87(2.1)32.7–41.0
3 months	26.47(4.3)17.8–35.1	26.00(5.0)16.1–35.9	27.92(4.1)19.7–36.1
With MDD			
Baseline	41.11(2.1)35.9–44.3	41.56(2.1)37.3–45.8	38.95(2.2)34.5–43.4
3 months	39.56(4.2)31.3–47.8	42.97(4.2)34.6–51.3	32.53(4.4)23.8–41.2

Covariates for the DASS-21 analysis were DASS-21 depression and physical component summary. Covariates for the IES-R analysis were IES-R intrusion and physical component summary

*CBT* cognitive behaviour therapy, *CI* confidence interval, *DASS-21* Depression, Anxiety and Stress Scale 21, *HL* healthy lifestyle, *IES-R* Impact of Events Scales (Revised), *MDD* major depressive disorder *SE* standard error of the mean

### Acceptability and attrition

Acceptability of the interventions appears to be positive. In the CBT group, 18 of 30 participants (60.00%) were satisfied or /very satisfied with the program, 11 (36.66%) were neither satisfied nor dissatisfied and 1 (3.33%) was very dissatisfied. For the HL group (two had missing data), 17 of 30 participants (56.66%) were satisfied or very satisfied, 8 (26.66%) were neither satisfied nor dissatisfied and 3 (10%) were dissatisfied. Similar acceptability of the CBT and HL modules was found. The participants also believed the program was worthwhile, with 22 of 30 (73.33%) CBT participants and 18 (60.00%) of HL participants responding positively. Finally, 21 of 30 (70.00%) CBT participants would recommend the CBT program, whereas 20 of 30 (66.66%) HL participants would recommend the HL program. Attrition rates were less than 20% (see Fig. 1).

### Feasibility

While recruitment into the RCT has been slow, the method and goal of recruitment are feasible. At this preliminary stage, 104 participants have been recruited with 14 dropouts, resulting in 30 per group. Slow recruitment is due in part to reluctance to participate in prospective research involving treatment soon after an MVC, especially in the context of sustaining a physical injury and being engaged in a potentially stressful compensation process. Figure 1 shows that at the time of analysis, 61% who met the inclusion criteria agreed to receive information about participation in the trial. Of those who received information, 65% consented to participate. Some participants gave a reason for non-consent: (1) no time to read modules, (2) excessive pain, (3) assistance not required and (4) legal advice against receiving assistance. These reflect the difficulty of recruiting through an insurer, and further, delays occurred due to frequent re-organisations of the insurance company and the limited time for case managers to introduce the research to potential participants. Recruitment within 4 months from MVC to claim lodgement proved problematic due to: (1) delays in achieving contact with participants and (2) the need for frequent reminders to provide consent and complete assessments. Table 1 shows that there were approximately 3 months (mean days = 82.09, standard deviation, SD = 57.08) from the MVC to completion of the baseline assessment. Completion of the post-intervention assessment often did not occur immediately after program completion, often taking several weeks and repeated phone calls (mean calls = 2.99, SD = 2.4). Further, the need for frequent reminders to complete assessments may have negatively influenced participants’ program feedback compared to their positive verbal feedback received during the intervention. Rapport ratings (Table 1) suggest more positive rapport was established with the CBT group than the HL group, with 28 of 30 (93.33%) positive ratings for CBT compared to 23 of 30 (76.67%) positive ratings for the HL group.

Finally, not all who met the inclusion criteria had access to email, resulting in some postal delivery of modules. Table 1 shows email was an efficient means of delivery with 84 (93.33%) of 90 participants receiving their modules by email, and only 6 (6.67%) having no access to email who received modules via the post.

## Discussion

The preliminary results demonstrate that brief email-delivered CBT or HL interventions with clinically focussed telephone support is most likely not required for participants without a diagnosis of MDD or PTSD, at least up to 3 months post-MVC. This conclusion is supported by the significantly reduced psychological distress over time regardless of group, shown in Figs. 2 and 3. For those diagnosed with MDD soon after their MVC, the findings shown in Fig. 5 offer rudimentary support for the efficacy of a brief CBT intervention delivered by email to reduce psychological distress as assessed by the IES-R. Nevertheless, Fig. 4 shows DASS-21 total results by group, suggesting benefit can be gained by either CBT or regularly calling people following an MVC who were engaged in seeking compensation and providing them with compensation-related reading material. In contrast, the benefits of providing a brief HL intervention for those with MDD are not promising and potentially harmful, given the results in Figs. 4 and 5 indicate that these participants did not show reduced distress over time. Whilst there was no change in MDD or PTSD diagnoses by 3 months, the major aim of reducing psychological distress appears to have been achieved for those without MDD, with positive signs of reduced distress in those with MDD in the CBT and control groups.

These preliminary findings are important, first because they have influenced the decision to cease recruitment to the HL intervention. Further, they provide possible direction for managing psychological distress in people sustaining MVC-related injuries who are engaged in seeking compensation; a situation identified as having a significant risk of elevated psychological distress and adverse outcomes, such as a delayed return to work [8], as well as lengthier and more costly claims [43]. With completion of the trial, further analysis of the total participant pool with 6- and 12-month post-MVC longitudinal data will help clarify the efficacy of the brief interventions. This is especially true for determining the usefulness of HL interventions for those diagnosed with MDD (or PTSD) soon after their MVC. For example, physically injured MVC survivors may be overwhelmed or irritated by an intervention that requests they monitor and improve their diet, sleep and exercise behaviour soon after a traumatic event, strategies that may be perceived to be unrelated to their injuries. Accordingly, the RCT will now continue as a two-arm trial, with the CBT and control groups.

Preliminary acceptability and feasibility results including adherence rates, delivery methodology and researcher-rated rapport all appear positive. However, the slow recruitment into the trial through the insurer partners has challenged an initial goal of the study, which was to recruit as early as possible after the MVC to help prevent the development of MDD and PTSD [11, 30]. On completion of the study, we intend to investigate whether those recruited earlier after the MVC (e.g. within 2 months) have superior outcomes than those recruited later (e.g. 3–4 months post-MVC).

### Study limitations

The 104 (30/30/30 with attrition) participants are more than likely a biased sample, given that fewer than 50% of the participants approached by the insurers agreed to enter the trial. Reasons for this include approaching participants at a stressful time, a perception of no need for psychological help and the opt-in style used for recruitment. It is also noted that the sample appears highly educated and the impact of all these limitations on bias in the sample needs to be considered when drawing conclusions. A further limitation relates to pre-morbid mental health history. This information is not routinely collected by insurance companies. However, the information collected about prior treatment by a psychologist or psychiatrist and on whether participants are taking prescribed psychiatric medications do provide a proxy for pre-morbid mental health history, which has been shown to be a strong predictor of post-MVC psychological distress [44, 45]. However, Table 1 showed pre-morbid psychological distress was not significantly different between the groups. We intend to explore fully the relationship between pre-morbid psychological distress and distress outcomes at the completion of the trial.

## Conclusions

This is the first study to investigate a brief email-delivered clinically focussed treatment offered to consenting MVC survivors irrespective of crash fault status or pre-morbid physical and mental health. Preliminary findings have identified the need to discontinue the HL intervention. The findings also offer preliminary guidance on improving mental health status for those with an MVC-related injury and engaged in seeking compensation. The limited compensation and health resources need to be directed to where they are needed most to ensure timely and effective recovery. Moreover, there is need to improve the cost-effectiveness of claims managed by insurance companies. For example, for those assessed as not meeting psychiatric criteria for MDD or PTSD, a beneficial long-term outcome may require only the provision of relevant claim material and a fortnightly telephone call, 3–6 months after seeking compensation. Further, for those identified with MDD, a cautious interpretation may be that the provision of brief CBT sessions early after an MVC may protect against chronic psychological disorders. The preliminary data suggest the RCT is feasible and acceptable and therefore, the study will continue to recruit and conduct follow-up assessments to 12 months post-MVC. More detailed analysis of the acceptability of the interventions and their potential associations with outcomes will be reported in future publications at the conclusion of the RCT.

## Additional file

Additional file 1: Consort checklist: recommended items to address in a clinical trial. (DOC 217 kb)

## Abbreviations

BMI: Body mass index
CBT: Cognitive behaviour therapy
CI: Confidence interval
DASS-21: Depression, Anxiety and Stress Scale 21
DSM-5: Diagnostic and Statistical Manual of Mental Disorders, 5th Edition
HL: Healthy lifestyle
IES-R: Impact of Events Scales (Revised)
MANCOVA: Multi-variate analysis of covariance
MDD: Major depressive disorder
MVC: Motor vehicle crash
NSW: New South Wales
PTSD: Post-traumatic stress disorder
RCT: Randomised controlled trial
SD: Standard deviation
SE: Standard error of the mean
SF: Short Form
